# Essential amino acid ingestion alters expression of genes associated with amino acid sensing, transport, and mTORC1 regulation in human skeletal muscle

**DOI:** 10.1186/s12986-017-0187-1

**Published:** 2017-05-11

**Authors:** Ted G. Graber, Michael S. Borack, Paul T. Reidy, Elena Volpi, Blake B. Rasmussen

**Affiliations:** 1 Division of Rehabilitation Sciences, University of Texas Medical Branch, 301 University Blvd, Galveston, TX 77555-1124 USA; 2 Department of Internal Medicine – Geriatrics, University of Texas Medical Branch, 301 University Blvd, Galveston, TX 77555-1124 USA; 3 Department of Nutrition and Metabolism, University of Texas Medical Branch, 301 University Blvd, Galveston, TX 77555-1124 USA; 4 Present Address: Department of Physical Therapy, University of Utah, Salt Lake City, Utah USA

**Keywords:** Muscle, Protein synthesis, mTORC1, Chloroquine, Lysosome

## Abstract

**Background:**

Amino acid availability stimulates protein synthesis via the mTORC1 (mechanistic target of rapamycin complex 1) signaling pathway. In response to an increase in cellular amino acid availability, translocation of cytosolic mTORC1 to the lysosomal surface is required to stimulate mTORC1 kinase activity. However, research elucidating the amino acid responsive mechanisms have thus far only been conducted in in vitro models.

Our primary objective was to determine whether an increase in amino acid availability within human skeletal muscle in vivo would alter the expression of genes associated with amino acid sensing, transport and mTORC1 regulation. Our secondary objective was to determine whether an acute perturbation in lysosomal function would disrupt the normal pattern of muscle amino acid responsive gene expression.

**Methods:**

We recruited 13 young adults into one of two groups: The first group ingested 10 g of essential amino acids (EAA). The second group ingested 10 g of EAA in the presence of chloroquine (CQ), a lysosomotropic agent. The subjects from each group had biopsies of the *vastus lateralis* taken before and after EAA ingestion. We determined the relative mRNA expression of 51 potential amino acid responsive genes using RT-qPCR.

**Results:**

There was a differential mRNA expression for 22 genes, with 15 mRNAs significantly changing (*P* < 0.05) in response to EAA ingestion (e.g., REDD1: +209 ± 35%; SLC38A9: +31 ± 9%; SLC38A10: +57 ± 15%). In the CQ group, EAA ingestion resulted in a differential expression as compared to EAA alone (i.e., 11 out of the 22 genes were different (*P* < 0.05) between the two groups.)

**Conclusions:**

Expression of several amino acid sensing, transport, and mTORC1 regulatory genes in human skeletal muscle are responsive to an increase in amino acid availability. Furthermore, potential acute disruption of lysosomal function by ingestion of chloroquine interferes with the normal pattern of gene expression following feeding. Our in vivo data in humans provide preliminary support for the in vitro work linking amino acid sensing pathways to mTORC1 translocation to the lysosome.

**Trial Registration:**

NCT00891696. Registered 29 April 2009.

**Electronic supplementary material:**

The online version of this article (doi:10.1186/s12986-017-0187-1) contains supplementary material, which is available to authorized users.

## Background

The mechanistic target of rapamycin complex 1 (mTORC1) is a master regulator of protein synthesis. mTORC1 exists as a dimer and consists of the core subunits mTOR, Raptor (regulatory-associated protein of mTOR), mLST8 (mammalian homolog of protein Lethal with Sec 13), and noncore regulatory subunits DEPTOR (DEP Domain Containing MTOR-Interacting Protein) and PRAS40 (Proline-rich AKT1 substrate 1) [1]. mTORC1 receives inputs from a variety of sources including amino acid availability, the presence or absence of growth factors, energy status and oxygen levels in the cell [2–5]. During anabolic conditions, mTORC1 has multiple outputs that include downregulation of autophagy (through TFEB, Transcription Factor EB, and ULK1, Unc-51 Like Autophagy Activating Kinase 1), and the triggering of mRNA translation and protein synthesis in part via phosphorylation of downstream effectors S6K1 (ribosomal protein S6 kinase 1) and 4E-BP1 (eukaryotic translation initiation factor 4E-binding protein) [6].

Amino acid availability is a potent anabolic stimulator of mTORC1 activity [4]. How cells sense an increase in intracellular amino acid availability is currently unknown, however, several groups have recently identified several mechanisms of amino acid sensing [7–10]. More than 50 proteins have been implicated in amino acid sensing. For example, the upstream (of mTORC1) proteins, Sestrin2 and CASTOR, have recently been identified as sensors of leucine and arginine, respectively [11, 12]. There is some controversy regarding the relationship between leucine and Sestrin2 [13]. Sestrin2 and CASTOR1 appear to regulate GATOR2/GATOR1 control of mTORC1 translocation to the lysosomal membrane where mTORC1 interacts with Rheb to activate mTORC1 kinase activity [11, 12, 14]. Several other proteins are also involved in controlling mTORC1 translocation to the lysosome and subsequent activation. These other proteins include an amino acid transporter located within the lysosome (SLC38A9), several GTPases (e.g., RagA/B, RagC/D), and scaffolding complexes (e.g., Ragulator) [8–10, 15, 16].

Much of the work characterizing amino acid sensing has used cultured HEK-293 T kidney cells as the model. In vitro work often does not translate to in vivo conditions, particularly in complex tissues such as skeletal muscle. Therefore, the primary aim of the current study was to determine whether genes known to regulate amino acid sensing in vitro would be responsive to an increase in amino acid availability in vivo. Previous work in muscle cells has shown a responsiveness of some amino acid transporter genes to amino acid feeding [17]. We hypothesized that an increase in amino acid availability within human skeletal muscle would alter the amino acid responsive gene expression profile. Furthermore, since the lysosome plays an integral role in the activation of mTORC1, as demonstrated in vitro [7], we also hypothesized that perturbing lysosomal function in humans would alter the normal post-prandial gene expression pattern in muscle. There is evidence that the mTORC1 downstream transcriptional activator TFEB (transcription factor EB) is activated not only with pharmacological and starvation induced inhibition of mTORC1, but also when lysosomal function is disrupted with the anti-malarial drug chloroquine [18]. We tested gene expression in this case rather than protein expression, because protein turnover, in most cases, is likely not measureable over the few hours between biopsies in this study. To test these hypotheses, we randomized young adults into two groups. Both groups ingested 10 grams of essential amino acids (EAA) while the second group also received the drug chloroquine (CQ; a lysosomotropic agent known to acutely disrupt lysosomal function) during hyperaminoacidemia. Muscle biopsies were collected before and after EAA ingestion to determine the change in mRNA expression of 51 genes associated with amino acid sensing, transport, and mTORC1 regulation.

## Methods

### Research design

Studies were conducted under protocols approved by the University of Texas Medical Branch Institutional Review Board, written informed consent was obtained from all subjects, in compliance with the Helsinki Declaration of 1975 as revised in 1983. All subjects self-reported during screening as having normal recreational levels of activity. Subjects reporting an exercise training regimen of more than 2× per week were excluded. DEXA (dual x-ray absorptiometry) assessed body composition. Exclusion criteria included: diabetes, any active diet or large weight loss, diseases of lung, liver, neurologic, blood, cardiovascular or kidney, history of cancer, current infectious disease, dementia or other significant psychiatric disease, and obesity. This work was a part of a larger study registered as NCT00891696 at clinicaltrials.gov; only information pertinent to the current study is imparted herein.

We recruited, screened and consented young adults into one of two groups: 10 g ingestion of essential amino acids (EAA: *n* = 6, 5 males and 1 female) or 10 g ingestion of EAA in the presence of chloroquine (CQ: *n* = 7, 4 males and 3 females). There was no difference (*P* > 0.05) in the age (EAA: 24.8 ± 1.3 years; CQ: 27.5 ± 1.7 years), BMI (body mass index, EAA: 22.1 ± 1.8; CQ: 22.7 ± 2.7) or body fat percentage (EAA 21.2 ± 1.8%; CQ 23.9 ± 2.7%) between the two groups.

The EAA composition we used was based on the known composition of high quality proteins (see Table 1).

**Table 1:** Composition of amino acid supplement

	% of total	Grams (g)
Histidine	11	1.1000
Isoleucine	10	1.0000
Leucine	18	1.8500
Lysine	16	1.5500
Methionine	3	0.3000
Phenylalanine	16	1.5500
Threonine	14	1.4500
Valine	12	1.2000

Chloroquine perturbs lysosome function by being preferentially sequestered within the lysosomal lumen where it absorbs protons thereby raising pH and causing dysfunction of the acid hydrosylases tasked to break up proteins, organelles and large peptides into recyclable amino acids. In addition, chloroquine also disrupts fusion of the autophagosome to the lysosome, thereby limiting substrate availability for the acid hydrolases [19, 20].

The evening prior to the study the participants received a standardized meal based upon their body mass and were then fasted overnight. Subjects in the CQ group received an oral dose of 250 mg of CQ the night before the study and then a second dose of 500 mg first thing in the morning on the study day (time = 0 h). The prescribed dosage of chloroquine (half-life of ~1–2 months) was the maximum allowed as defined by the study physician, was similar to the therapeutic dosage used to treat malaria, and was consistent with a previous study on protein metabolism [18, 21, 22]. The subjects from each group had biopsies of the *vastus lateralis* taken after 4 h of resting, at time = 4 h after the administration of the second chloroquine dose for the CQ group. This period was considered the basal or (pre-EAA) period. Following the muscle biopsy, each subject ingested the same 10 g of EAA. One hour after EAA ingestion, a second muscle biopsy was obtained (post-EAA), as this time point coincides with peak EAA availability and maximal mTORC1 signaling.

### Tissue collection

We obtained vastus lateralis muscle biopsies pre- and post-intervention using aseptic techniques, as previously described [23]. Briefly, following the use of 1% lidocaine as a local anesthetic and subsequent incision with a disposable sterile scalpel, we used a 5-mm Bergstrom biopsy needle to collect the biopsy. Both the pre-EAA biopsy and post-EAA biopsy were from the same leg. Collected muscle tissue was immediately flash frozen in liquid nitrogen and then stored at −80 °C until needed for assays.

### RNA isolation and cDNA production

We performed RNA isolation and cDNA production similarly to what has been previously reported by our lab [17]. Briefly, we homogenized frozen tissue in 1 ml of Tri-reagent (Molecular Research Center, P/N TR 118). RNA was then isolated following the manufacturer’s directions and quantified for concentration using a Nanodrop 2000. The mean concentration obtained was 319.3 ng/μl. The mean 260/280 ratio was 1.89 and the mean 260/230 ratio was 1.52. The integrity of the RNA was assessed using an Agilent 2100 BioAnalyzer (Agilent Technologies) using the manufacturer’s instructions, with a mean integrity of 7.94 ± 0.01 (scale 1–10, 10 being perfect) and the mean 28S-to-18S ratio at 1.35 ± 0.004. The RNA was then treated with deoxyribonuclease (DNase) (Applied Biosystems DNA-free kit, #AM1906), following the manufacturer’s instructions, to remove any endogenous genetic DNA. Then 3 μg of purified RNA underwent the reverse transcriptase reaction to produce cDNA (Bio-Rad iScript kit, #170-8891), following the manufacturer’s instructions, scaled to a reaction volume of 60 μl, using a BioRad iQ5 to run the reaction (protocol: 25 °C for 5 min; 42 °C for 30 min and 85 °C for 5 min). The cDNA was stored at −80 °C.

### PrimePCR plates

We designed five different custom 96-well plates that were prepared and validated/optimized in house by BioRad (validation data searchable for each primer in the PrimePCR section at www.bio-rad.com). Each plate contained 10 target genes and 2 reference genes (β2-microgobulin, B2M, and Glyceraldehyde 3-phosphate dehydrogenase, GAPDH). The target genes (all associated with mTORC1 signaling and/or amino acid sensing/transport) were: SLC38A1, SLC38A2, SLC38A4, SLC38A5, SLC38A6, TFEB, SLC38A7, SLC38A8, SLC38A9, SLC38A10, DDIT4, DDIT4L, LAMP2, MAP1LC3A, RHEB, SLC36A1, SLC36A2, SLC7A1, SLC7A5, SLC7A8, ATP6VOD1, C7orf59, HBXIP, LAMTOR1, LAMTOR2, LAMTOR3, RRAGA, RRAGB, RRAGC, RRAGD, DEPDC5, FLCN, FNIP1, MIOS, NPRL2, NPRL3, SEC13, SEH1L, WDR24, WDR59, AKT1S1, DEPTOR, LARS, MLST8, mTOR, PIK3C3, RPTOR, TBC1D7, TSC1, and TSC2. There was no difference in expression of the two reference genes (B2M and GAPDH) from the basal to fed condition (*P* > 0.20).

### Sestrin2 (SESN2)

The SESN2 (SESTRIN2) and the two reference genes, B2M and GAPDH, primers were self-designed and purchased from SIGMA. See Table 2 for details. All primers showed a single peak on the melt curves; had r^2^ values of 0.9854, 1.000 and 0.9970 on the efficiency curve of the dilution series, and had efficiencies of 106, 90, and 105%, respectively for SESN2, B2M and GAPDH. Neither reference gene changed in response to the interventions (*P* > 0.2) and was considered stable.

**Table 2:** Primer details (SESN2, B2M and GAPDH)

Gene	Sequence (5′- > 3′)	Length	Start	Stop	Tm	GC%	Length	Accession #
SESTRIN2	FWD GCCACTCAGAGAAGGTCCAC	20	1739	1758	60.04	60		NM_001199933.1
	REV: GAGTCAGGTCATGTAGCGGG	20	1842	1823	59.9	60	104	Exon 1752/1753 fwd
B2M	FWD: CCAAAGATTCAGGTTTACTCAC	22			52.5	40.9		NM_00404
	REV: TCAACTTCAATGTCGGATGG	20			52.8	45	101	
GAPDH	FWD: AGGTGAAGGTCGGAGTCAAC	20			56.3	55		J02642
	REV: GCTCCTGGAAGATGGTGATG	20			55	55	231	

### RT-qPCR

We determined the relative mRNA expression of the 51 potentially amino acid responsive genes using RT-qPCR (BioRad CFX) on pre-EAA and post-EAA muscle biopsy samples from 11 of the 13 study participants (*n* = 6 for CQ and 5 for EAA, as adequate tissue was not obtained from 2 of the enrolled female subjects in the EAA group). For the PrimePCR plates: 10 μl of SYBR green supermix (BioRad, P/N 172–5271), 3 μl of template (from 1:8 dilution of cDNA with DNase/RNase free water), and 9 μl RNase-free water were loaded into wells, in duplicate for each subject sample, on the PrimePCR plate. The plate was sealed, spun down on a centrifuge, and then loaded into the CFX cycler. The following protocol was used: 1 cycle for 2 min @ 95 °C; then 40 cycles of: 95 °C for 5 s, 60 °C for 30 s; followed by 95 °C for 10 s and a melt curve. Results were quantified using CFX connect software (BioRad) and then the 2^-∆∆Ct^ method was used on the C_q_ (quantification cycle) to calculate the final mRNA fold-change values from pre-EAA to post-EAA using the geometric mean of the two reference genes and reported according to MIQE guidelines [24–26]. We performed SESTRIN2 RT-qPCR in a similar fashion, with B2M and GAPDH as reference genes: 12.5 μl of SYBR green supermix (BioRad, P/N 172–5271), 3 μl of template (1:8 dilution), 8.3 μl RNase-free water, 00.7 μl forward template for SESN2 and 0.5 μl reverse template were loaded into wells, in duplicate for each subject sample. The protocol used on the CFX cycler was: 1 cycle for 3 min @ 95 °C; then 40 cycles of: 95 °C for 10 s, 55 °C for 30 s; followed by 95 °C for 10 s and a melt curve.

### Statistics

Data is presented as means ± SE, or as % change ± SE, as appropriate. Student’s paired *T*-test (within groups testing the difference between the two time points, before and after EAA ingestion within subjects) and 2x2 Repeated Measures ANOVA (comparisons between the two groups, EAA and CQ, at the two time points, before and after EAA ingestion) used *p* < 0.05 for significance, with trends reported at *p* < 0.10. Statistical software package was SPSS v.23 (IBM).

## Results

### Overview

In order to determine the effect of EAA ingestion on gene expression, we measured the expression levels of 51 genes involved in processes including amino acid sensing, amino acid transport and mTORC1 regulation. There were significant changes in 22 of the 50 genes tested from pre- to post-EAA. In the normal feeding scenario (EAA), expression of 15 mRNAs changed: SLC38A10, SLC38A9, C7orf59, WDR24, MIOS, DDIT4, DPEDC5, NPRL2, TSC1, AKT1S1, MLST8, mTOR, RPTOR, PIK3C3 and TFEB all increased (*P* < 0.05). In EAA, 6 more also had increases: SLC7A8, NPRL3, TSC2, SLC36A1, LAMTOR2, and MAP1LC3; but had *p*-values *P* < 0.10 and >0.05--heretofore referred to as genes of interest. In the group that ingested EAA and chloroquine (CQ), 6 mRNAs changed expression: SLC38A10, SLC7A1, TBC1D7, and TSC2 all decreased (*P* < 0.05); while DDIT4L and NPRL3 both increased (*P* < 0.05). CQ had 4 genes of interest: SLC38A9, DEPDC5 and LAMP2 increased while LAMTOR3 decreased (*P* < 0.10 and >0.05). There were significant differences (*P* < 0.05) between the fold changes in the two treatment groups in 11 different genes (SLC38A10, SCL7A8, WDR25, DDIT4, NPRL2, TSC2, AKT1S1, mTOR, RPTOR and TFEB all being expressed to a greater extent in EAA, with only DDIT4L being expressed higher in CQ). In addition, between the groups there were trend (*P* < 0.10 and >0.05) differences in 7 other genes: SLC7A1, TBC1D7, TSC1, SLC38A4, LAMTOR1, LAMTOR2, and FLCN all showed a greater fold change in EAA. The genes differentially expressed and the genes of interest coded for a variety of different mRNAs that translate into proteins/protein complexes that are both positive and negative regulators of mTORC1 activity (20), amino acid transporters (6), autophagy genes (2), mTORC1 subunits (4), and lysosomal formation/maintenance proteins (2) (some mRNAs belong to more than one category). Additional file 1: Table S1 (S signifying in the supplement) summarizes the significant findings with the percent change from pre-EAA to post-EAA, the name and function of the protein/protein complex coded for by the mRNA, and associated *p*-values. Additional file 1: Table S2 in the supplement summarizes the genes of interest in the same format. In all figures, the female subjects (*n* = 3 in CQ) are delineated by darker data points.

### Specific gene responses

#### Amino acid transporters

**Fig. 1:**
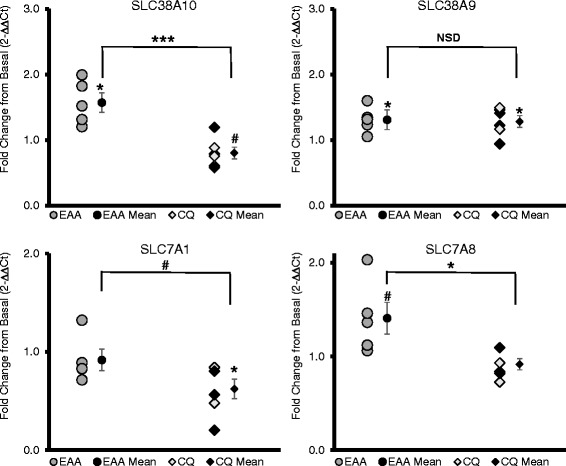
Amino Acid Transporters. SLC38A10 and SLC38A9 are both sodium-coupled neutral amino acid transporters, with SLC38A9 implicated in activating RAGULATOR and hence mTORC1 and SLC38A10 having an unknown function. SLC7A1 is a cationic transporter (e.g., arginine) and SLC7A8 codes for the LAT2 light subunit. x-axis: EAA = essential amino acid only group, CQ = essential amino acid plus chloroquine group, mean = mean of group ± standard error; y-axis units is fold change in gene expression from the basal to fed state using the 2-∆∆Ct method; bar indicates independent *t*-test comparison between EAA and CQ, symbols over means indicate paired *t*-test comparison of fold change with normalized baseline; * = *p* ≤ 0.05, ** = *p* ≤ 0.01, # = *p* < 0.10, NSD or no label = *p* > 0.10; the female subjects (*n* = 3 in CQ) are delineated by darker data points

Out of 14 amino acid transporters and subunits examined, 4 were *significantly* affected by the treatments (*P* < 0.05). SLC38A10 increased 57 ± 15% in EAA, but decreased −20 ± 9% in CQ; SLC38A9 increased 31 ± 9% in EAA; SLC7A1 decreased −37 ± 11% in CQ; and both SLC7A8 and SLC38A10 had higher expression in EAA than CQ (see Fig. 1).

#### GATOR complex (GATOR1 and GATOR2)

**Fig. 2:**
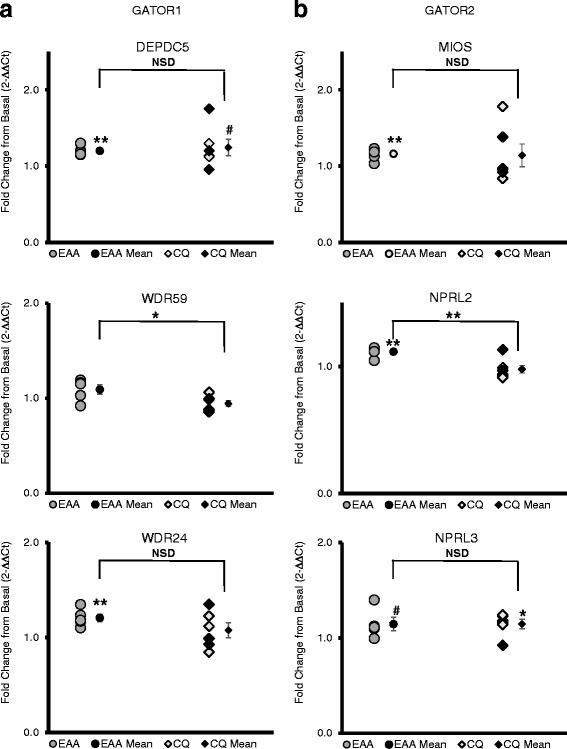
GATOR Complex. **a** GATOR1 is a negative regulator of mTORC1, acting as a GAP towards Rag A/B. **b** GATOR2 is a positive regulator of mTORC1, acting as a negative regulator of GATOR1. x-axis: EAA = essential amino acid only group, CQ = essential amino acid plus chloroquine group, mean = mean of group ± standard error; y-axis units is fold change in gene expression from the basal to fed state using the 2-∆∆Ct method; bar indicates independent *t*-test comparison between EAA and CQ, symbols over means indicate paired *t*-test comparison of fold change with normalized baseline; * = *p* ≤ 0.05, ** = *p* ≤ 0.01, # = *p* < 0.10, NSD or no label = *p* > 0.10; the female subjects (*n* = 3 in CQ) are delineated by darker data points

The GATOR complex accounted for changes in 6 of the 22 genes affected overall. Three of the five GATOR1 mRNAs increased (*P* < 0.05): DEPDC5 20 ± 3% and NPRL2 12 ± 2% in EAA, and NPRL3 15 ± 5% in CQ. All three of the mRNAs coding genes from the GATOR2 complex (positive regulator of mTOC1) showed a significant change (*P* < 0.05): MIOS 16 ± 4% and WDR24 21 ± 4% increasing in EAA, and WDR59 higher in EAA than CQ (see Fig. 2).

#### Negative regulators of mTORC1

**Fig. 3:**
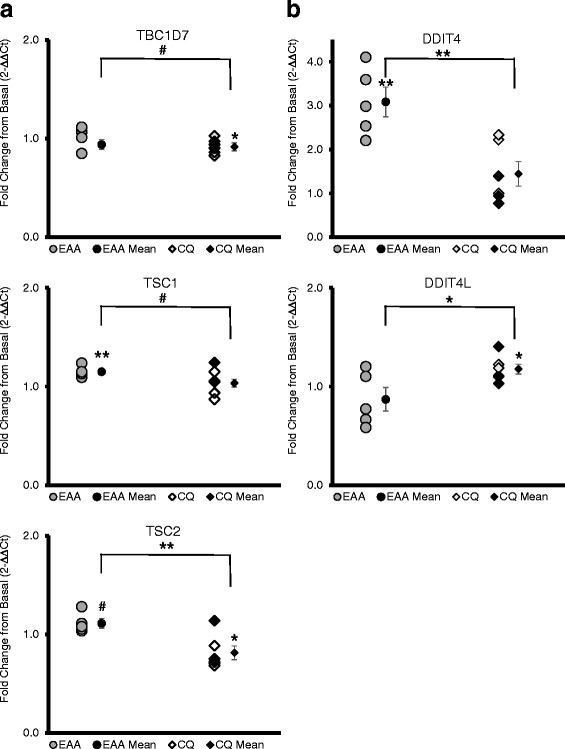
**a** TSC Complex. The TSC complex has 3 subunits, TSC1, TSC2 and TBC1D7. TSC1 increased in EAA, but TSC2 and TBC1D7 decreased in CQ. The TSC complex is a negative regulator of mTORC1, acting as a negative regulator of Rheb. **b** REDD1 and REDD2. REDD1 and REDD2 have the same function, negatively regulating mTORC1 through the TSC complex. The mRNAs coding for REDD1 (DDIT4) and for REDD2 (DDIT4L) changed in an opposite manner. x-axis: EAA = essential amino acid only group, CQ = essential amino acid plus chloroquine group, mean = mean of group ± standard error; y-axis units is fold change in gene expression from the basal to fed state using the 2-∆∆Ct method; bar indicates independent *t*-test comparison of EAA and CQ, symbols over means indicate paired *t*-test comparison of fold change with normalized baseline; * = *p* ≤ 0.05, ** = *p* ≤ 0.01, # = *p* < 0.10, NSD or no label = *p* > 0.10; the female subjects (*n* = 3 in CQ) are delineated by darker data points

TSC (tuberous sclerosis complex) subunits’ mRNA expression was differentially expressed by treatment, with TSC1 increasing 15 ± 2% in EAA, and TSC2 (−19 ± 7%) and TBC1D7 (−8 ± 3%) decreasing in CQ (Fig. 3a). Genes coding for the REDD1 and REDD2 proteins were different (*P* < 0.05) between EAA and CQ, with DDIT4 (REDD1) mRNA expression increasing 209 ± 35% in EAA and DDIT4L (REDD2) mRNA increasing by 18 ± 5% in CQ (Fig. 3b). In addition, AKT1S1 (coding for PRAS40, an mTORC1 inhibitory subunit) mRNA expression increased by 29 ± 7%, as did RPTOR 27 ± 9% (coding for RAPTOR, another conditionally mTORC1 inhibitory subunit) (Additional file 1: Figure S1).

#### mTOR/Autophagy/Ragulator

The mRNA expression of four mTORC1 subunits (AKT1S1 mentioned above, MLST8 16 ± 5%, mTOR 30 ± 10%, RPTOR 27 ± 9%) increased (*P* < 0.05) after EAA ingestion in the EAA group, but were not changed (*P* > 0.05) in the CQ group (Additional file 1: Figure S1). There were also significant increases in two genes associated with autophagy in EAA: PIK3C3 14 ± 4% and TFEB 96 ± 19%, and in one that codes for a subunit of RAGULATOR, C7orf59 (i.e., LAMTOR4) 18 ± 6% (Additional file 1: Figure S2) [27].

#### SESTRIN2

There was no change is SESN2 mRNA expression from pre to post EAA ingestion in the CQ group (percent change was −6.5 ± 5.9, *P* = 0.32), but there was a trend for a 63.7 ± 29.0% increase in SESN2 mRNA expression in the EAA group, *P* = 0.09. There was quite a bit of variance in the EAA group with 3 subjects having ~2-fold increases in expression and 2 non-responders. The EAA group had a trend for a higher mRNA expression of SESN2 in response to EAA in comparison with the CQ group (*P* = 0.071). See Additional file 1: Figure S3 in the supplement for details.

## Discussion

**Fig. 4:**
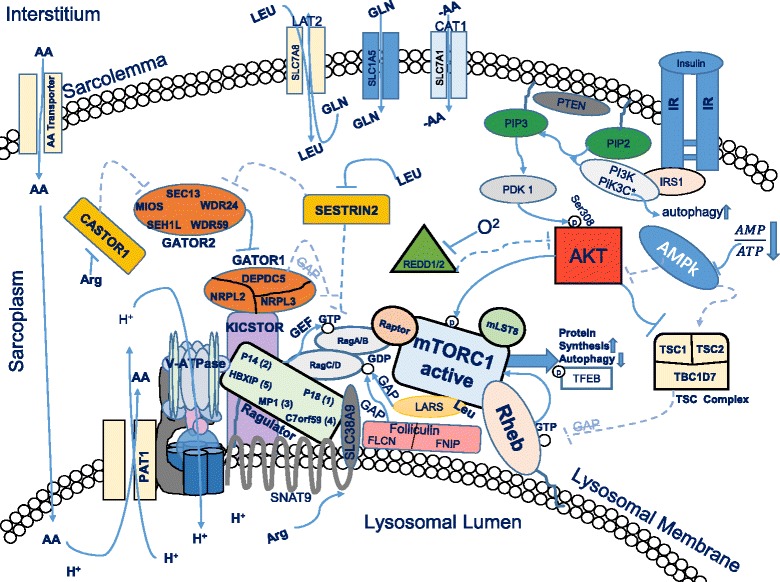
Simplified Schematic of mTORC1 Regulation. In the post-prandial state an increase in amino acid and insulin availability results in Akt phosphorylation by PDK1 at serine 308, and Redd1/2 and the TSC complex are inhibited. Under these conditions, Rheb is active at the lysosomal membrane and can, in turn, activate mTORC1 kinase activity. With sufficient amino acids in the cell, the Rag heterodimer is recruited to the lysosome where it interacts with Rheb to increase mTORC1 kinase activity. This results in increased protein synthesis and inhibition of autophagy via phosphorylation of S6K1 and 4E-BP1, and ULK1 and TFEB, respectively. In the current study, EAA ingestion in the group that did not receive chloroquine, we found that the following proteins/protein complexes depicted in the schematic had increased mRNA expression (*p* < 0.05 or *p* < 0.10): SNAT9, CAT1, LAT2, PAT1, SESTRIN2, REDD1, TSC-TSC2 complex, Ragulator, GATOR1 and GATOR2, PI3K, mTORC1, and TFEB. For a complete listing of the genes and how the conditions of the study changed mRNA expression, refer to Additional file 1: Tables S1 and S2 in the supplement. AA = amino acids, LEU = leucine, ARG = arginine, GLN = glutamine, GAP = GTPase activating protein, GEF = guanine exchange factor, GTP = guanine triphosphate, GDP = guanine diphosphate. H + =hydrogen ion (proton), O2 = oxygen. Text within shapes = protein or subunit names. *Dotted lines* and *arrows* indicate repressed functions under the stated conditions

We found that a total of 22 genes related to amino acid sensing, amino acid transport and mTORC1 regulation in human skeletal muscle changed significantly as a result of the ingestion of 10 grams of essential amino acids in the EAA group (see Additional file 1: Table S1), with another 11 genes showing a trend (See Additional file 1: Table S2). Furthermore, administration of chloroquine prior to EAA ingestion altered the gene expression pattern. For example, the CQ group had no change in 16 of the 22 genes exhibiting a change (significant or trend) in the EAA group (Additional file 1: Tables S1, S2). In the CQ group there was a differential expression of 5 mRNAs (i.e., significantly changed in the CQ group but with no change in the EAA group), and a trend for a differential expression in 2 genes that did not change in the EAA group. There were 11 mRNAs showing a significant difference between the two groups (EAA and CQ) and 7 more mRNAs exhibiting a trend for a group difference. For reference, we have provided a simplified schematic of the signaling pathways displaying some of the relationships between the proteins coded for by the genes we examined (Fig. 4). Both hypotheses are supported by the data: 1) an increase in amino acid availability in human skeletal muscle increases the expression of genes associated with amino acid sensing, as well as other genes we term as amino acid responsive and 2) administration of chloroquine to alter lysosomal function results in a differential pattern of gene expression.

### Amino acid transporter mRNA expression post-EAA

We examined the expression levels of numerous amino acid transporters due to their close relationship with protein synthesis and mTORC1 signaling. In response to an increase in amino acid availability both SLC38A10 and SLC38A9 mRNA expression increased in the EAA group, with both SLC38A10 and SLC7A1 (cationic amino acid transporter, CAT1) decreasing in the CQ group. Both SLC38A9 and SLC38A10 are sodium-coupled neutral amino acid transporters, with SLC38A9 serving the function as lysosomal lumen arginine sensor contributing to activation of RAGULATOR. SLC38A10 has not been fully characterized, but is believed to have a similar function [28]. SLC7A8 (LAT2 light subunit) trended to increase by 41% ± 17, (*P* = 0.077) in EAA, but the difference between EAA and CQ was significant (49%, *P* = 0.018). LAT2 is a neutral amino acid antiporter that is similar to LAT1 in that it pumps out nonessential amino acids (glutamine) and pumps in neutral essential amino acids (leucine), acting as a critical regulator of leucine concentration in the cell. The only difference in LAT1 and LAT2 is in the composition of the light subunit, as both also contain the heavy subunit CD98, with the LAT2 subunit (SLC7A8) having a broader substrate range [29].

Previous work discovered gene expression of some amino acid transporters significantly increased at 1-h post-EA [23]. These transporters included SLC7A5 (LAT1 light subunit, LAT = L-Type amino acid transporters), SLC3A2 (CD98, heavy chain subunit of the L-type transporters), A-type amino acid transporter SLC38A2 (SNAT2; SNAT = sodium-coupled neutral amino acid transporter) and proton assisted amino acid symporter SLC36A1 (PAT1). The current study did not detect significant increases in SLC7A5 or SLC38A2. This is in part due to large individual variability: 29 ± 30 and 36 ± 30%, with coefficients of variation = 232 and 186, respectively. However, we found that SLC36A1 (PAT1) trended toward an increase (27 ± 11%, *P* = 0.063, due to the presence of 1 negative outlier: without the outlier it increased 37 ± 5%, *P* = 0.005). Therefore, while there were some differences in mRNA expression of particular transporters from prior studies, the important thing to note is that a number of transporters were affected by an increase in amino acid availability, suggesting that the amino acid transport system is sensitive not only to changes in amino acid concentrations, but also to the functional status of the lysosome.

### Regulators of mTORC1

Previous work has established the role of both amino acid sensing and nutritional regulation in mTORC1 activation [4, 5, 8]. Out of the 23 positive regulators of mTORC1 examined, only 5 increased with EAA. These include the SLC38A9 arginine sensor discussed above in the amino acid transporter section, 3 different subunits of GATOR2 (WDR24, WDR25 and MIOS) and the RAGULATOR subunit LAMTOR4, C7orf59. Six of the 20 genes associated with mTORC1 regulation (both positive and negative) that changed in EAA or CQ code for the GATOR1/2 complex proteins. In the cytosol, GATOR1 (GATOR = GAP, GTPase Activating Protein, activity towards Rag) consists of the subunits DEPDC5 (DEP domain-containing protein 5), NPRL2 (Nitrogen Permease Regulator 2-Like Protein) and NPRL3 (Nitrogen Permease Regulator 3-Like Protein). GATOR1 inhibits Rag A/B with GAP (keeping it in the low energy inactive GDP state) [12, 30]. Thus, GATOR1 interacts with the lysosome indirectly as an mTORC1 inhibitor via Rag A/B GAP activity, while regulated by GATOR2. GATOR2 consists of 5 subunits: MIOS (WD repeat-containing protein mio), WDR24 (WD repeat-containing protein 24), WDR59 (WD repeat-containing protein 59), SEH1L (Nucleoporin SEH1) and SEC13 (Protein SEC13 homolog), and is involved in activating mTORC1 upon amino acid availability. SESN2 mRNA expression, upstream of mTORC1 and GATOR1/2, tended to be increased by 60% in the EAA group with no change in the CQ group. Thus, it appears that amino acid availability directly affects gene expression of SESN2 and the GATOR complex associated genes.

### Other negative regulators of mTORC1

TSC complex genes increased in EAA (TSC1), but decreased in CQ (TSC2 and TBC1D7). In addition, AKT1A1 (PRAS40) and DDIT4 (REDD1) mRNA expression also increased in the EAA group. Low oxygen levels in the cell are detected via Redd1 (DDIT4; DNA-Damage-Inducible Transcript 4) or Redd2 (DDIT4L; DNA-Damage-Inducible Transcript 4-like protein), activating the TSC complex (consisting of 3 SUBNUITS: TSC 1, TSC 2, and TBC1D7--TBC1 domain family member 7). There is evidence that REDD1 and REDD2 act to modulate mTORC1 signaling by adjusting gain to limit output and that the proteins are responsive to both nutrient levels and exercise [31]. The TSC complex inhibits Rheb (Ras Homolog Enriched In Brain) via GAP (GTPase activating protein) activity. GAP increases the hydrolysis rate of GTP to GDP. Rheb is active only in the high energy GTP state and necessary for stimulating mTORC1 kinase activity [32]. Additionally, some evidence suggests that the TSC complex also acts to sterically hinder the Rheb kinase site and may be recruited to the lysosome by Rag A/B during conditions of low amino acid availability in the cell [33].

More negative regulators (10) of mTORC1 activity increased with EAA than positive regulators (5). The upregulation of negative regulator genes may indicate the existence of a negative feedback loop.

### Limitations

There are a few limitations with our study that should be discussed. First, the total amount of chloroquine that was administered was relatively low. This was done in order to ensure participant safety. However, this may have resulted in an incomplete inhibition of lysosomal function. Also, since we did not independently measure lysosomal activity, we can only assume that lysosomal function was perturbed at least partially by chloroquine. Second, we did not measure protein expression as it is unlikely that we would be able to detect a change in protein expression within the one-hour time frame of our intervention. In addition, much of the transient changes in mTORC1 activation is driven by covalent modification of proteins rather than by overall protein content. The changes in mTORC1 signaling post-amino acid ingestion has been previously well documented [2, 4–7, 9, 15, 27], thus, since this work focused on gene expression changes, we did not repeat that work in this study. Finally, while we did delineate between male and female subjects in the figures, we and others have previously shown that signaling and protein synthesis, both at baseline and in response to essential amino acids, is not sex dependent [34–36].

## Conclusions

In our in vivo study, we provide preliminary evidence to support our hypothesis that a discernable pattern of gene expression occurred in response to an acute increase in amino acid availability. In addition, the normal gene expression response to amino acid availability is partially disrupted by the administration of chloroquine. In fact, in 19 of the 22 genes found to have been significantly altered, the administration of CQ resulted in a differential mRNA expression pattern (i.e., mRNA expression did not change or changed in the opposite direction of the EAA group). This finding highlights the importance of the lysosome in amino acid response in the in vivo setting. We acknowledge that mRNA expression at a single time point is only a snapshot of a dynamic system. Based upon these findings, future work may confirm the link between amino acid response and mTORC1 translocation to the lysosome. Thus, future mechanistic work in muscle cells and in transgenic mice are required to determine definitively whether the amino acid sensing mechanisms identified in vitro are functional in skeletal muscle.

## Additional file

Additional file 1: Table S1. Gene Expression Results. **Table S2.** Genes of Interest (*p* < 0.1). **Figure S1.** mTORC1 Subunits. **Figure S2.** Other Genes (PDF 639 kb).

## Abbreviations

BMI: Body mass index
cDNA: Complementary DNA
CQ: Chloroquine
DEXA: Dual x-ray absorptiometry
EAA: Essential amino acid
RT-qPCR: Real time quantitative polymerase chain reaction
SE: Standard error
