# Supplementary material for: Essential amino acid ingestion alters expression of genes associated with amino acid sensing, transport, and mTORC1 regulation in human skeletal muscle
Source: Nutr Metab (Lond). 2017 May 11;14:35. doi: 10.1186/s12986-017-0187-1 (PMC5426042; doi:10.1186/s12986-017-0187-1)
Supplement: Additional file 1: Table S1. — Gene Expression Results. Table S2. Genes of Interest (p < 0.1). Figure S1. mTORC1 Subunits. Figure S2. Other Genes (PDF 639 kb). [file 12986_2017_187_MOESM1_ESM.pdf]

Essential Amino Acid Ingestion Alters Expression of Genes Associated with Amino Acid Sensing, Transport, and mTORC1 Regulation in Human Skeletal Muscle

Online Supplementary Materials

Table S1 Gene Expression Results

Table S2 Genes of Interest (0.05< p <0.1)

Figure S1 mTORC1 Subunits

Figure S2 Other Genes

Figure S3 SESN2

Essential Amino Acid Ingestion Alters Expression of Genes Associated with Amino Acid Sensing, Transport, and mTORC1 Regulation in Human Skeletal Muscle

		**Differential Gene Expression Data**	**EAA**		**CQ**		**EAA vs. CQ**	
**Gene**	**Protein/Complex Name**	**Protein Function**	**%Change[1]**	**p-val.[2]**	**%Change[1]**	**p-val.[2]**	**%dif.[3]**	**p-val.[4]**
		**Amino Acid Transporters and Subunits**						
**SLC38A10**	**SNAT10**	sodium-dependent amino acid/proton antiporter	**57 ± 15**	**0.019**	**-20 ± 9**	**0.001**	**77**	**0.001**
**SLC38A9**	**SNAT9**	arginine sensor upstream of ragulator	**31 ± 9**	**0.025**	*24 ± 10*	*0.058*	6	0.645
**SLC7A1**	**ATRC1 (CAT1)**	cationic amino acid transporter 1	-8 ± 11	0.473	**-37 ± 11**	***0.016***	*29*	*0.081*
**SLC7A8**	**LAT2**	neutral amino acid antiporter light subunit	*41 ± 17*	*0.077*	-8 ± 6	0.241	**49**	**0.018**
		**Positive Regulators of mTORC1**						
**C7orf59**	**Ragulator**	aka: LAMTOR4, GEF to RAG A/B	**18 ± 6**	**0.042**	5 ± 10	0.632	13	0.216
**WDR24**	**GATOR2**	Inhibits GATOR1	**21 ± 4**	**0.007**	8 ± 8	0.373	13	0.194
**WDR59**	**GATOR2**	Inhibits GATOR1	9 ± 5	0.15	-6 ± 3	0.152	**15**	**0.034**
**MIOS5**	**GATOR2**	Inhibits GATOR1	**16 ± 4**	**0.012**	14 ± 15	0.393	2	0.904
		**Negative Regulators of mTORC1 and mTORC1 Subunits**						
**DDIT4**	**REDD1[5]**	cellular oxygen sensing, modulates Akt	**209 ± 35**	**0.004**	44 ± 28	0.170	**164**	**0.005**
**DDIT4L**	**REDD2[5]**	cellular oxygen sensing, modulates Akt	-13 ± 12	0.331	**18 ± 5**	**0.022**	**-31**	**0.035**
**DEPDC5**	**GATOR1[5]**	GAP to RAG A/B	**20 ± 3**	**0.002**	*24 ± 11*	*0.080*	-4	0.738
**NPRL3**	**GATOR1[5]**	GAP to RAG A/B	*14 ± 7*	*0.098*	**15 ± 5**	**0.028**	-1	0.993
**NPRL2**	**GATOR1[5]**	GAP to RAG A/B	**12 ± 2**	**0.003**	-2 ± 3	0.538	**14**	**0.007**
**TBC1D7**	**TSC1-TSC2[5]**	GAP to Rheb;	2 ± 5	0.364	**-8 ± 3**	**0.039**	*11*	*0.078*
**TSC1**	**TSC1-TSC2[5]**	GAP to Rheb;	**15 ± 2**	**0.003**	3 ± 5	0.548	*12*	*0.092*
**TSC2**	**TSC1-TSC2[5]**	GAP to Rheb;	*11 ± 4*	*0.062*	**-19 ± 7**	**0.049**	**30**	**0.008**
**AKT1S1**	**PRAS40[5]**	mTORC1 subunit--kinase inhibitor	**29 ± 7**	**0.014**	4 ± 8	0.647	**26**	**0.037**
**MLST8**	**MLST8**	mTORC1 subunit	**16 ± 5**	**0.007**	1 ± 7	0.884	15	0.129
**mTOR**	**mTOR**	serine/threonine kinase mTORC subunit	**30 ± 10**	**0.044**	'-6 ± 8	0.465	**36**	**0.020**
**RPTOR**	**RAPTOR**	scaffold for recruiting mTORC1 substrates	**27 ± 9**	**0.041**	'-4 ± 4	0.368	**31**	**0.009**
		**Important for Autophagy**						
**PIK3C3**	**PI3K**	mediates formation of PIP3, autophagasome	**14 ± 4**	**0.026**	6 ± 6	0.401	8	0.345
**TFEB**	**TFEB**	Transcription factor positive for autophagy	**96 ± 19**	**0.007**	-8 ± 15	0.614	**104**	**0.002**

Table S1 Gene Expression Results. Bold indicates significant p-value (**p<0.5**), *Italics* indicate a trend *(p<0.1)*, EAA indicates group fed essential amino acids, CQ indicates group fed essential amino acids while under the influence of chloroquine, EAA vs. CQ indicates a comparison between the changes seen in both groups. [1] percent change from basal to fed condition, [2] p-value from 2-tail paired student's t-test, [3] percent difference (EAA %change - CQ% change), [4] p-value taken from 2x2 Repeated Measures ANOVA between-subjects comparison by group, [5] a negative regulator of mTORC1.

**Genes of Interest (p<0.10)**			**EAA**		**CQ**		**EAA vs. CQ**	
**Gene**	**Protein Name**	**Protein Function**	**%Change[1]**	**p-val.[2]**	**%Change[1]**	**p-val.[2]**	**%dif.[3]**	**p-val.[4]**
		**Amino Acid Transporters and Subunits**						
**SLC38A4**	**SNAT4**	sodium-dependent neutral amino acid transporter	114 ± 59	0.126	-2 ± 11	0.847	*116*	*0.063*
**SLC36A1**	**PAT1**	amino acid symporter; positive regulator of mTOR	*27 ± 11*	*0.07*	20 ± 21	0.381	7	0.792
		**Positive Regulators of mTORC1**						
**LAMTOR1**	**Ragulator**	aka: p18; anchors RAGULATOR to lysosome	15 ± 12	0.147	-4 ± 4	0.414	*19*	*0.06*
**LAMTOR2**	**Ragulator**	aka: p14	*21 ± 9*	*0.071*	-0.4 ± 6	0.942	*22*	*0.059*
**LAMTOR3**	**Ragulator5**	aka: MP1	14 ± 20	0.533	*-11 ± 5*	*0.061*	25	0.226
**FLCN**	**Folliculin**	Folliculin subunit, GAP towards RAG C/D	149 ± 76	0.144	11 ± 14	0.459	*138*	*0.058*
		**Important for Lysosome**						
**LAMP2**	**LAMP2**	lysosome maintenance of acidic environment	26 ± 18	0.228	*18 ± 9*	*0.098*	8	0.681
**MAP1LC3**	**LC3A**	lysosome membrane formation	*54 ± 21*	*0.064*	35 ± 24	0.207	18	0.59

Table S2 Genes of Interest (p<0.1)

Italics indicate a trend (*p<0.1*), EAA indicates group fed essential amino acids, CQ indicates group fed essential amino acids while under the influence of chloroquine, EAA vs. CQ indicates a comparison between the changes seen in both groups. [1] percent change from basal to fed condition, [2] p-value from 2-tail paired student's t-test, [3] percent difference (EAA %change - CQ% change), [4] p-value taken from 2x2 Repeated Measures ANOVA between-subjects comparison by group.

Essential Amino Acid Ingestion Alters Expression of Genes Associated with

Amino Acid Sensing, Transport, and mTORC1 Regulation in Human Skeletal Muscle

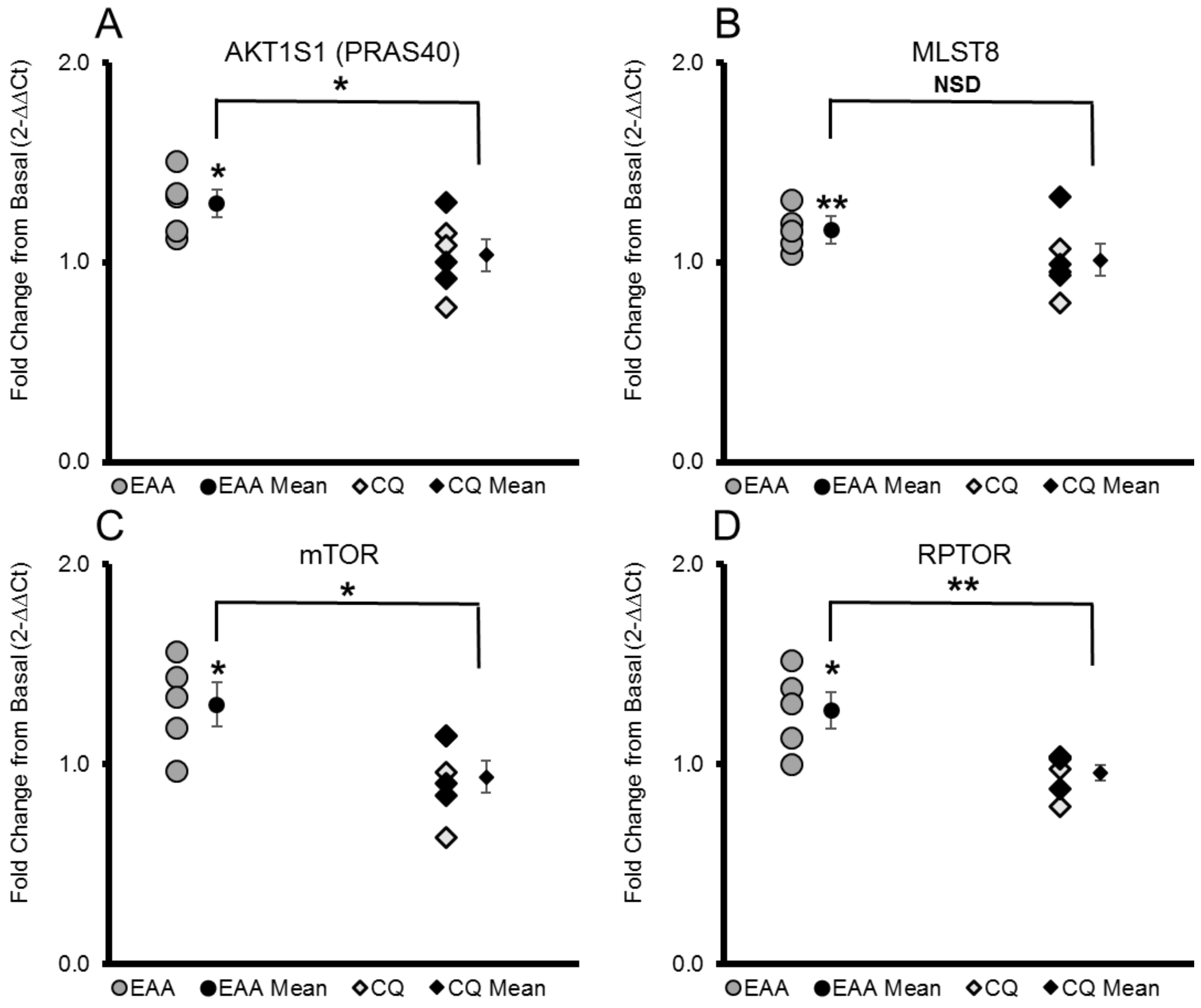

Figure S1 mTORC1 Subunits. Genes for four subunits of mTORC1 were upregulated in EAA, but not CQ, including: **A**) AKT1S1 (regulatory subunit PRAS40); **B)** MLST8 gene; **C)** serine/threonine kinase subunit gene mTOR; **D**) RPTOR (regulatory RAPTOR). x-axis: EAA = essential amino acid only group, CQ = essential amino acid plus chloroquine group, mean = mean of group ± standard error; y-axis units is fold change in gene expression from the basal to fed state using the 2-ΔΔCt method; bar indicates independent t-test comparison between EAA and CQ, symbols over means indicate paired t-test comparison of fold change with normalized baseline; * = $p \leq 0.05$, ** = $p \leq 0.01$, # = $p < 0.10$, NSD or no label = $p > 0.10$; the female subjects (n=3 in CQ) are delineated by darker data points.

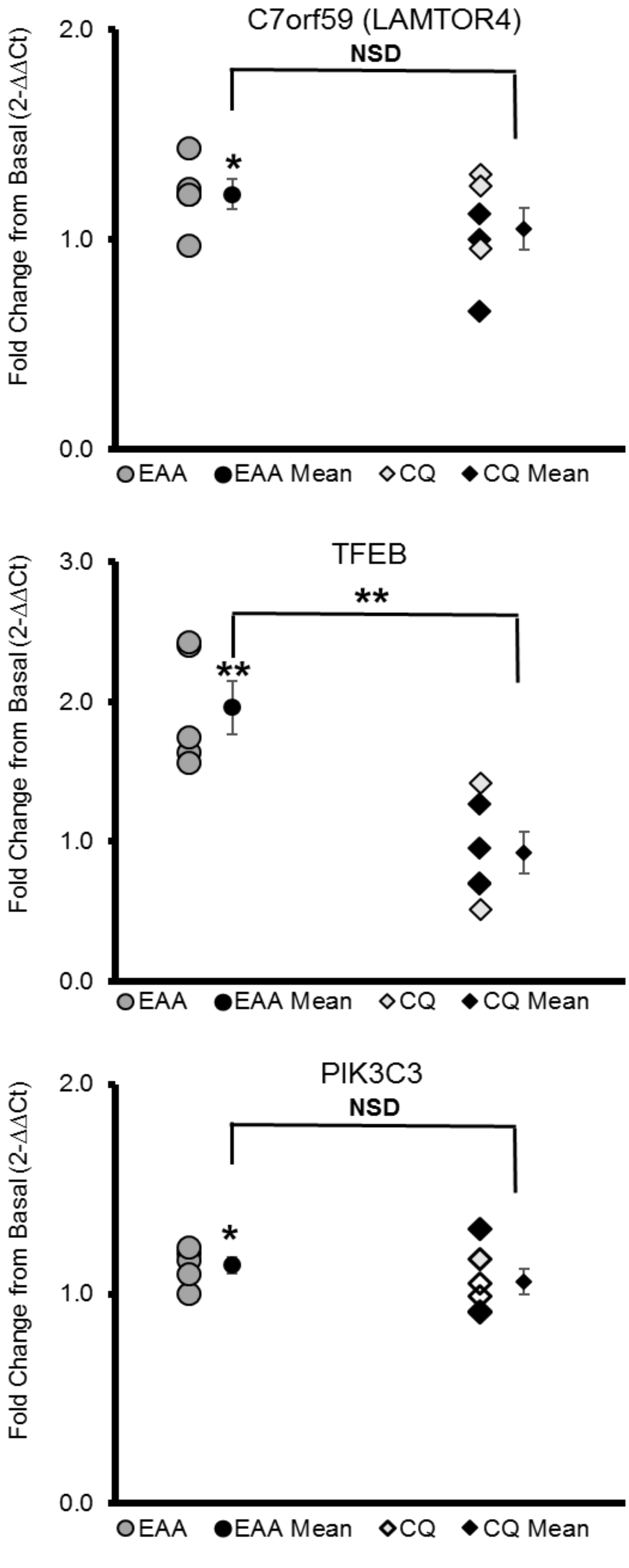

Figure S2 Other Genes. Autophagy related genes TFEB and PIK3C3 increased in EAA but not in CQ, as did the RAGULATOR subunit mRNA for LAMTOR4, C7orf59. x-axis: EAA=essential amino acid only group, CQ=essential amino acid plus chloroquine group, mean = mean of group ± standard error; y-axis units is fold change in gene expression from the basal to fed state using the 2-ΔΔCt method; bar indicates independent t-test comparison between EAA and CQ, symbols over means indicate paired t-test comparison of fold change with normalized baseline; * = $p \leq 0.05$, ** = $p \leq 0.01$, # = $p < 0.10$, NSD or no label = $p > 0.10$; the female subjects (n=3 in CQ) are delineated by darker data points.

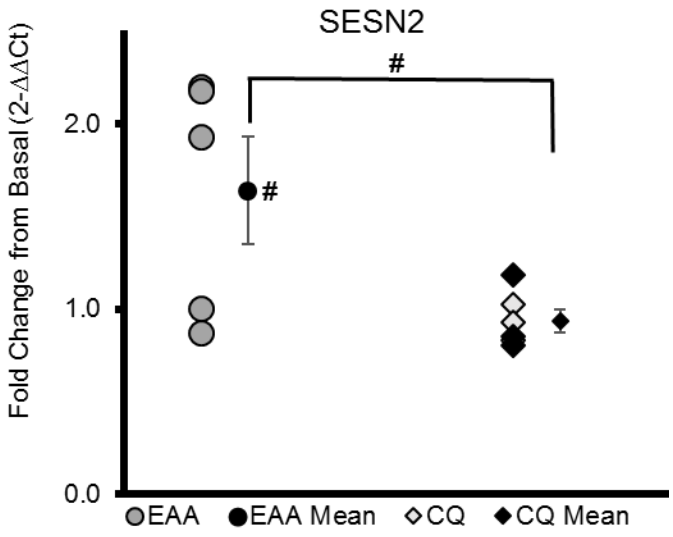

Figure S3 SESN2.
SESN2 gene codes for the Sestrin2 protein, one function of which is to act to inhibit GATOR2 (thus inhibiting mTORC1). Sestrin2 is deactivated with sufficient leucine. x-axis: EAA=essential amino acid only group, CQ=essential amino acid plus chloroquine group, mean = mean of group ± standard error; y-axis units is fold change in gene expression from the basal to fed state using the 2-ΔΔCt method; bar indicates independent t-test comparison between EAA and CQ, symbols over means indicate paired t-test comparison of fold change with normalized baseline; * = $p \leq 0.05$, ** = $p \leq 0.01$, # = $p < 0.10$, NSD or no label = $p > 0.10$; the female subjects (n=3 in CQ) are delineated by darker data points.
